# Iodinated contrast media inhibit oxygen consumption in freshly isolated proximal tubular cells from elderly humans and diabetic rats: Influence of nitric oxide

**DOI:** 10.3109/03009734.2016.1144664

**Published:** 2016-02-29

**Authors:** Per Liss, Peter Hansell, Angelica Fasching, Fredrik Palm

**Affiliations:** a Department of Oncology, Radiology and Clinical Immunology, Section of Radiology, University Hospital, Uppsala, Sweden; b Department of Medical Cell Biology, Section of Integrative Physiology, Uppsala University, Uppsala, Sweden; c Department of Science and Health, Division of Drug Research, University of Linköping, Linköping, Sweden

**Keywords:** Contrast media, human, nitric oxide, oxygen consumption, proximal tubule, rat

## Abstract

**Objectives:**

Mechanisms underlying contrast medium (CM)-induced nephropathy remain elusive, but recent attention has been directed to oxygen availability. The purpose of this study was to evaluate the effect of the low-osmolar CM iopromide and the iso-osmolar CM iodixanol on oxygen consumption (QO_2_) in freshly isolated proximal tubular cells (PTC) from kidneys ablated from elderly humans undergoing nephrectomy for renal carcinomas and from normoglycemic or streptozotocin-diabetic rats.

**Materials:**

PTC were isolated from human kidneys, or kidneys of normoglycemic or streptozotocin-diabetic rats. QO_2_ was measured with Clark-type microelectrodes in a gas-tight chamber with and without each CM (10 mg I/mL medium). L-NAME was used to inhibit nitric oxide (NO) production caused by nitric oxide synthase.

**Results:**

Both CM reduced QO_2_ in human PTC (about –35%) which was prevented by L-NAME. PTC from normoglycemic rats were unaffected by iopromide, whereas iodixanol decreased QO_2_ (–34%). Both CM decreased QO_2_ in PTC from diabetic rats (–38% and –36%, respectively). L-NAME only prevented the effect of iopromide in the diabetic rat PTC.

**Conclusions:**

These observations demonstrate that CM can induce NO release from isolated PTC *in vitro*, which affects QO_2_. Our results suggest that the induction of NO release and subsequent effect on the cellular oxygen metabolism are dependent on several factors, including CM type and pre-existing risk factors for the development of CM-induced nephropathy.

## Introduction

Contrast medium (CM)-induced nephropathy (CIN) is the third leading cause of acute renal failure in patients who have been admitted to the hospital, accounting for 10% of all cases (1,2). The underlying causes of CIN remain to be established, but several mechanisms have been suggested, with hypoxic insults to the renal medulla dominating the arena (3,4). Due to the vascular architecture of the renal medulla with countercurrent exchange vessels (*vasa recta*) the oxygen availability is low already during normal physiological conditions. Furthermore, conditions closely associated with the development of kidney damage (hypertension and diabetes) display even further reduced oxygen availability in the kidney tissue (5–7). Pre-existing renal impairment is a well-established risk factor for the development of CIN. It has therefore been suggested that CM may cause medullary hypoxia which results in the development of CIN. Most likely, a combination of various mechanisms is responsible for the development of CIN (4), but the pathophysiological relevance of direct effects of CM on tubular cells is contentious (8), as are the other proposed etiologies (4,9).

It should be noted that hypoxia per se is fairly well tolerated by most cells as long as the energy requirements are kept low, e.g. reduced metabolism due to cooling. However, adenosine triphosphate (ATP) deprivation as a result of either decreased mitochondrial oxygen utilization or inhibition of the mitochondrial electron transport chain (and, thus, ATP production) is a far more severe condition, which activates several pathways in order to counteract the reduced ATP supply (10). It has been shown that nitric oxide (NO) is an important regulator of renal blood flow, i.e. oxygen delivery, but also directly affects the oxygen availability for regulating the mitochondrial oxygen utilization (11–13). It is well known that the medulla is the major site of CM-induced injury, but we used proximal tubular cells (PTC) in our study to investigate the effects of the different CM. There are two main reasons for choosing this approach: PTC can be isolated from human kidneys in the relatively high quantity needed for measurements of oxygen consumption (QO_2_), and they represent a cell type of high metabolic activity due to high electrolyte transport similar to the cells of the medullary thick ascending limb of the loop of Henle. The last-mentioned is believed to be the site of CM-induced injury.

The purpose of our study was to evaluate the effect of the low-osmolar CM iopromide and the iso-osmolar CM iodixanol on QO_2_ in freshly isolated PTC from kidneys of nephrectomized elderly humans, and kidneys from normoglycemic or streptozotocin-diabetic rats.

## Materials and methods

The Institutional Review Board for Uppsala University approved all protocols involving patients, and informed written consent was obtained from all patients before being enrolled in the study. All animal experiments were performed in accordance with the NIH guidelines for use and care of laboratory animals and approved by the local Animal Care and Use Committee. All chemicals were from Sigma-Aldrich (St. Louis, MO, USA) and of highest grade available if not otherwise stated.

### Patient data

Body weight, length, and blood pressure were recorded. Venous blood samples were collected in vacutainers (Kronans Droghandel, Stockholm, Sweden) containing gel/Li-heparin for analysis of plasma creatinine, containing gel for analysis of serum C-peptide, and containing EDTA for analysis of HbA_1c_ the day before surgery. The patients had been admitted to the hospital for unilateral nephrectomy due to renal carcinoma.

The kidney donors (*n* = 7) allocated to this study displayed characteristics typically for patients at increased risk to develop CIN, including decreased GFR, increased blood pressure, and increased body mass index (Table 1). Each donor was separately studied and analyzed.

**Table 1.** Patient characteristics (*n* = 7).

Age (years)	63.7 ± 4.3
Plasma creatinine (mmol/L)	85 ± 7
Calculated GFR (mL/min)	82 ± 10
Serum C-peptide (nmol/L)	2.64 ± 0.45
HbA_1c_ (%)	5.20 ± 0.13
Systolic blood pressure (mmHg)	147 ± 6
Diastolic blood pressure (mmHg)	86 ± 3
Body mass index (kg/m^2^)	27.6 ± 1.0

### Isolation of human proximal tubular cells

Human PTC were isolated according to a modified procedure originally described by Hawksworth (14), resulting in a cell suspension containing more than 95% PTC. In brief, a macroscopically healthy ablated part of the nephrectomized kidney (1–8 g tissue), as determined by the surgeon, was placed in ice-cold balanced salt solution (BBS) (in mmol/L: 5.37 KCl, 0.44 KH_2_PO_4_, 137 NaCl, 0.34 Na_2_HPO_4_, 1.35 NaHCO_3_, 5.56 D-glucose, 25 HEPES, 0.5 EGTA, 0.5% BSA, 50 mg/L streptomycin; pH 7.2). The kidney cortex was dissected under microscope (10× magnification) and chopped coarsely with a scalpel, transferred to new ice-cold BBS, and centrifuged at 100*g* for 2 min. The pellet was resuspended in DMEM/Ham’s F12 nutrient mixture (1:1, containing 15 mmol/L HEPES, 14.28 mmol/L NaHCO_3_, 50 mg/L streptomycin), and the centrifugation was repeated three times. The final pellet was resuspended in 37 °C DMEM/DMEM/Ham’s F12 containing 0.4% (w/v) collagenase A (*Clostridium histolyticum*, 0.5 U/mg) and incubated for 70 min at 37 °C. The cell suspension was then cooled on ice and filtered (pore sizes of 180, 75, 53, and 38 μm), pelleted by centrifugation (400*g*, 4 min), and finally resuspended in collagenase-free DMEM/Ham’s F12. This procedure was repeated three times prior to analysis of QO_2_. PTC from each patient were treated as *n* = 1 in the subsequent calculations of oxygen consumption.

### Induction of diabetes in rats

Adult male Sprague-Dawley rats (Scanbur, Sollentuna, Sweden) had free access to tap water and standard rat chow (R3, Ewos, Södertälje, Sweden). Diabetes was induced by a single injection of streptozotocin (55 mg/kg) in the tail vein. Animals were considered diabetic if the blood glucose concentrations increased to ≥15 mmol/L within 24 hours and remained elevated. Blood glucose concentrations were determined with test reagent strips (MediSense, Bedford, MA, USA) from blood samples obtained from the cut tip of the tail.

The body weight of the age-matched control group (*n* = 8) was 493 ± 8 g versus 313 ± 13 g for the diabetic group (*n* = 8), and the blood glucose concentrations were 7.3 ± 0.1 versus 29.7 ± 0.7 mmol/L, respectively. Each rat was studied and analyzed separately.

### Isolation of rat proximal tubular cells

The buffer solution had, if not stated otherwise, the following composition in mmol/L: 113.0 NaCl, 4.0 KCl, 27.2 NaHCO_3_, 1.0 KH_2_PO_4_, 1.2 MgCl_2_, 1.0 CaCl_2_, 10.0 HEPES, 0.5 Ca^2+^-lactate, 2.0 glutamine, and osmolality adjusted to 300 ± 2 mOsm/kg H_2_O, and pH to 7.40. Streptomycin (VWR International, Stockholm, Sweden) was added, resulting in a final concentration of 50 U/mL. For non-diabetic rats the buffer contained 5.8 mmol/L glucose, and for diabetic animals the buffer contained 23.2 mmol/L glucose (similar to the blood glucose concentrations in these latter animals). Two weeks after the induction of diabetes, PTC were isolated as previously described (7,12,15). In brief, the rats were anesthetized with thiobutabarbital and kidneys removed, placed on ice, and the cortex dissected. The cortical tissue was minced through a metallic mesh-strainer and immediately placed in an ice-cooled buffer solution containing 0.05% (w/v) collagenase. The minced tissue was incubated at 37 °C, while the buffer was equilibrated with 95% O_2_/5% CO_2_ and stirred manually every fifth min. The cell suspension was cooled on ice and filtered through graded filters (pore sizes 180, 75, 53, and 38 μm) and then treated similarly as human PTC.

### Measurements of oxygen consumption in vitro

QO_2_ was measured as previously described (7,12). A custom-made thermostatically controlled (37 °C) gas-tight plexiglas chamber with a total volume of 1.10 mL was used. A modified Unisense 500 O_2_ sensing electrode (Unisense, Aarhus, Denmark), calibrated with air-equilibrated buffer solution set to 228 μmol/L O_2_ and Na_2_S_2_O_5_-saturated buffer set to zero, measured O_2_ consumption. After calibration, 100 μL of cell suspension was injected into the chamber, and the rate of O_2_ disappearance was recorded and adjusted for protein concentration (DC Protein Assay; Bio-Rad Laboratories, Hercules, CA, USA). In addition to baseline QO_2_ measurements, cells were pre-incubated for 30 min with either the NO synthase inhibitor Nω-nitro-L-arginine-methyl-ester (L-NAME; 10 μg/mL), iopromide (10 mg I/mL; 590 mOsm/kg H_2_O; Schering AG, Berlin, Germany), iodixanol (10 mg I/mL; 290 mOsm/kg H_2_O; GE Healthcare, Stockholm, Sweden), or a combination of L-NAME and iopromide or iodixanol. The CM concentration during the QO_2_ measurements was calculated to be equal to the highest concentration PTC would be exposed to *in vivo* during a clinical examination. L-NAME is the most commonly used inhibitor of all three NO synthase isoforms. Each measurement of QO_2_ was performed in duplicate, and the average reported as *n* = 1.

### Western blot analysis

Isolated PTC were mixed 1:1 with lysis buffer (1.0% NP40, 0.5% sodium deoxycholate, 0.1% SDS, 10 mM NaF, 80 mM Tris; pH 7.5) containing enzyme inhibitors (Complete Mini; 1 tablet/3 mL; Roche Diagnostics, Mannheim, Germany). Total homogenates from rat brain were used as positive controls. Samples were run on 7.5% Tris-HCl gels with Tris/glycine/SDS buffer. Proteins were detected, after transfer to nitrocellulose membranes, using rabbit anti-nNOS (2 μg/mL; Zymed Laboratories, Invitrogen, Carlsbad, CA, USA), rabbit anti-eNOS (1:2000; Sigma Aldrich), and HRP-conjugated secondary goat anti-rabbit antibody (1:5000; Sigma-Aldrich) by an ECL-camera (Kodak image station 2000; New Haven, CT, USA).

### Statistics

All values are means ± SEM. Multiple comparisons within the same group were performed using repeated measures ANOVA followed by Dunnett’s test for paired comparisons (Statview, Abacus Concepts, Berkeley, CA, USA). Unpaired Student’s *t* test was used when comparing two data sets. *P* < 0.05 was considered statistically significant.

## Results

### Oxygen consumption of human PTC

**Figure 1.**
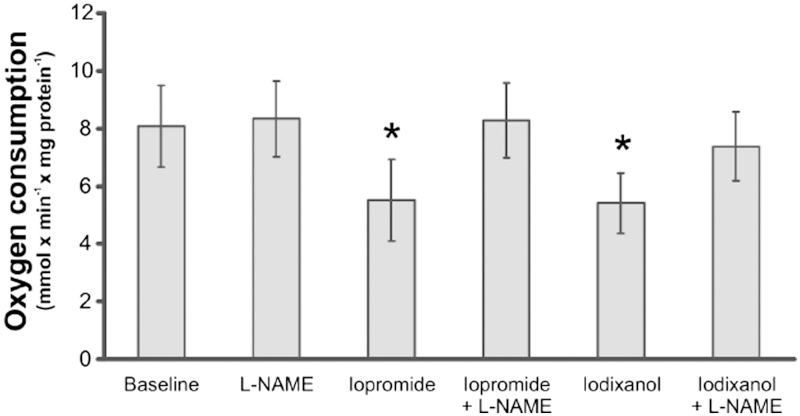
Oxygen consumption of freshly isolated human proximal tubular cells before and after incubation with the non-specific nitric oxide synthase inhibitor L-NAME (10 μg/mL), the contrast media iopromide (10 mg I/mL) or iodixanol (10 mg I/mL), or a combination (*n* = 7 per group). *P > 0.05 versus baseline. All values are means ± SEM.

Isolated human PTC were unaffected by L-NAME alone, whereas both iopromide and iodixanol significantly reduced QO_2_ (Figure 1). In the presence of L-NAME the effects of both iopromide and iodixanol oxygen consumption were abolished.

### Oxygen consumption of rat PTC

**Figure 2.**
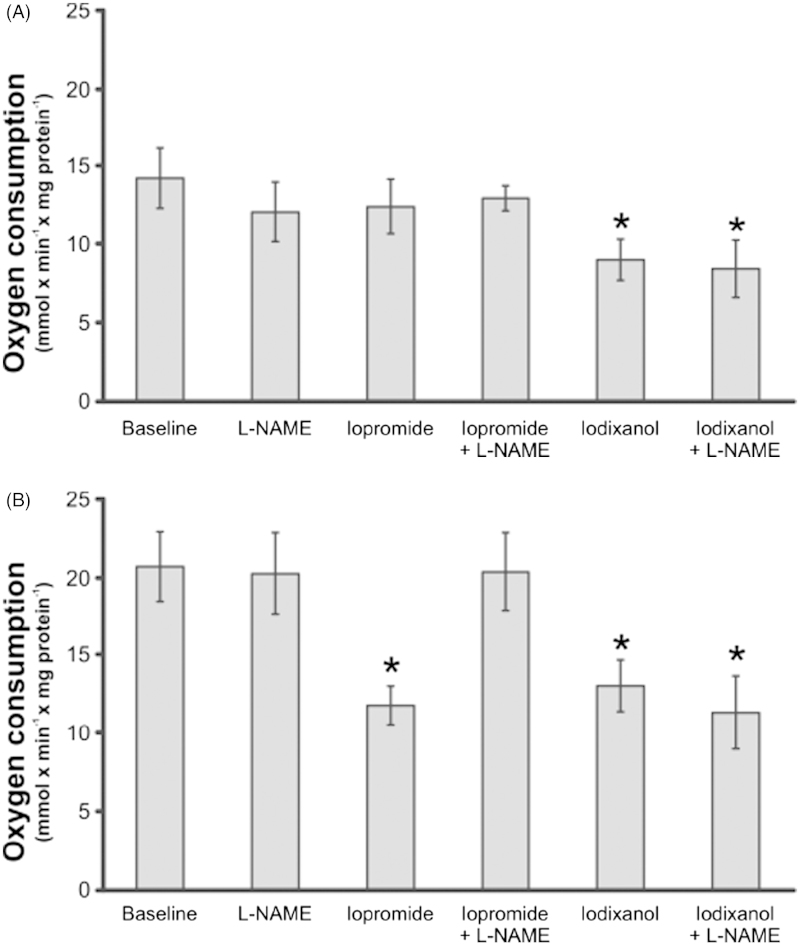
Oxygen consumption of freshly isolated rat proximal tubular cells isolated from control (A) or diabetic rats (B). Cells were incubated with the non-specific nitric oxide synthase inhibitor L-NAME (10 μg/mL), the contrast media iopromide (10 mg I/mL) or iodixanol (10 mg I/mL), or a combination (*n* = 8 per group). * *P* > 0.05 versus baseline within the same group. All values are means ± SEM.

PTC isolated from diabetic rats had increased baseline QO_2_ compared with corresponding controls (Figure 2). PTC prepared from normoglycemic control rats were unaffected by L-NAME, iopromide, or a combination of the two (Figure 2A). However, iodixanol reduced their QO_2_, and L-NAME did not prevent this reduction. L-NAME alone had no effect, whereas iopromide and iodixanol reduced their QO_2_ when PTC prepared from diabetic rats were examined (Figure 2B). L-NAME completely inhibited the effect of iopromide, but failed to prevent the effect of iodixanol.

### Protein identification

**Figure 3.**
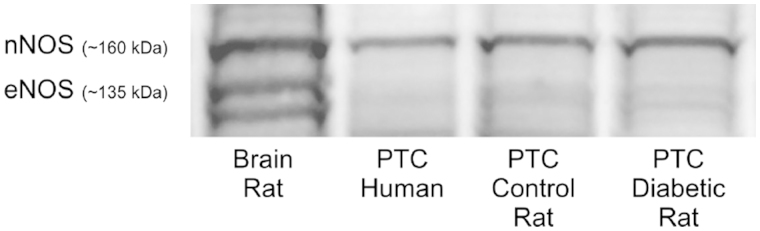
Western blot directed against the neuronal (n) and endothelial (e) nitric oxide synthases confirming the presence of NO-producing enzyme in isolated human and rat proximal tubular cells.

Western blot directed against nNOS and eNOS revealed the presence of nNOS in both human and rat PTC (Figure 3), in the latter case irrespectively of whether the cells had been prepared from diabetic or non-diabetic animals. Presence of eNOS was, however, not possible to document.

## Discussion

Our study demonstrated that there is reduced QO_2_ when freshly isolated human PTC were exposed to CM. Inhibition of NO production from NO synthase by L-NAME completely prevented this effect, indicating that CM induce NO release which in turn inhibits QO_2_. A similar response was seen in PTC from diabetic rats exposed to iopromide. However, PTC isolated from control rats were unaffected by iopromide. Although iodixanol reduced QO_2_ in PTC isolated from either control or diabetic rats, this effect was independent of NO synthase-derived NO since L-NAME did not prevent this effect. These novel findings demonstrate a new possible pathway in which CM can affect kidney epithelial cells directly via induction of NO release from NOS, which may influence cellular ATP production with consequences for cellular energetics and function. Together with previous studies showing that the delivery of oxygen also is compromised, the results provide a novel mechanism for the development of CIN.

It is well established that NO inhibits QO_2_ at the level of the mitochondria (11,12,16). CM-induced NO release from the intracellular NOS will reduce QO_2_ in the mitochondria. In turn, ATP production may be reduced. If this also occurs *in vivo* it may constitute a serious problem which will be added to other functional problems such as reduced renal blood flow and GFR (17,18). However, diabetic rats in a previous study did not display decreased cortical blood flow following iopromide administration, but rather a slight increase (19). This indicates that iopromide given to diabetic rats induces vasodilatation, which is in agreement with the findings in our study where iopromide induced a significant NO release by PTC isolated from human and diabetic rat kidneys.

It has previously been reported that NO is reduced in kidneys of diabetic rats, which increases QO_2_ resulting in reduced oxygen availability in the kidney (13,20). Tissue hypoxia has indeed been recognized as a unifying pathway to chronic kidney disease (20). It might therefore be contradictory that CM-induced NO release and subsequently reduced QO_2_ could be damaging. However, a vast majority of the energy requirement of the kidney (about 80%) is devoted to active electrolyte reabsorption of filtered electrolytes. A vasodilation concomitant to reduced ATP production would therefore place extra strain on an already vulnerable organ.

Why do normal human kidney cells display reduced QO_2_ in response to iopromide but not the corresponding rat cells? It is plausible that our PTC which were isolated from older human subjects cannot be considered as true ‘control’ cells corresponding to the rat control cell preparations. We do not want to overstate our risk factors in the patient material, but they may very well contribute to the results. The mean age of the nephrectomized patients was 61 years, and it is established that kidney function declines with age and is an independent risk factor for CIN. Furthermore, the patients had hypertension and reduced calculated GFR which also constitute risk factors for CIN. Sustained diabetes together with pre-existing renal impairment is also a strong independent risk factor for the development of CIN, and this might be the explanation for the similar results of our human PTC to that of the diabetic rat PTC.

We confirmed that nNOS is the dominating isoform expressed in PTC. This is in agreement with a previous report by Deng et al. (21). They showed that the QO_2_ by isolated rat PTC is inhibited by NO, and that the highly selective nNOS inhibitor S-methyl-L-thiocitrulline prevents the NO inhibition. The results of our study show that, in PTC isolated from elderly humans, both iopromide and iodixanol can induce NO release from nNOS, which in turn inhibits QO_2_. Iopromide induced a similar NO-dependent inhibition of the QO_2_ in the diabetic PTC. However, iodixanol inhibited QO_2_ of PTC prepared from both diabetic and control rats, and this effect could not be blocked by NOS inhibition. Whether or not CM also can induce NO release from the other NOS isoforms remains to be investigated. The differences in the mechanisms involved in reducing QO_2_ by the two different CM investigated in our study are unknown, and were not investigated further. It is, however, known that differences in CM properties affect kidney function differently (4).

Our study demonstrates that iodinated CM inhibits cellular QO_2_ in PTC isolated from elderly humans and rats, which can be due to CM-induced release of NO. However, the induction of NO release and subsequent effects on the cellular oxygen metabolism are dependent on several factors, including CM type and if the PTC originate from a patient with known pre-existing risk factors for CIN.

## Disclosure statement

P. Liss has previously held a grant by Bayer AG. The remaining authors declare no conflict of interest.

## Funding information

Funding for the project was received from the Swedish Research Council Medicine and Health, the Swedish Diabetes Foundation, and the Swedish Medical Association.
